# Engaged followership and toxic science: Exploring the effect of prototypicality on willingness to follow harmful experimental instructions

**DOI:** 10.1111/bjso.12603

**Published:** 2022-11-16

**Authors:** Megan E. Birney, Stephen D. Reicher, S. Alexander Haslam, Niklas K. Steffens, Fergus G. Neville

**Affiliations:** ^1^ School of Health, Science and Wellbeing Staffordshire University Stoke‐on‐Trent UK; ^2^ School of Psychology and Neuroscience University of St Andrews St Andrews UK; ^3^ School of Psychology University of Queensland Brisbane Australia; ^4^ School of Management University of St Andrews St Andrews UK

**Keywords:** followership, identity, Milgram, obedience

## Abstract

Drawing on the ‘engaged followership’ reinterpretation of Milgram's work on obedience, four studies (three pre‐registered) examine the extent to which people's willingness to follow an experimenter's instructions is dependent on the perceived prototypicality of the science they are supposedly advancing. In Studies 1, 2 and 3, participants took part in a study that was described as advancing either ‘hard’ (prototypical) science (i.e., neuroscience) or ‘soft’ (non‐prototypical) science (i.e., social science) before completing an online analogue of Milgram's ‘Obedience to Authority’ paradigm. In Studies 1 and 2, participants in the neuroscience condition completed more trials than those in the social science condition. This effect was not replicated in Study 3, possibly because the timing of data collection (late 2020) coincided with an emphasis on social science's importance in controlling COVID‐19. Results of a final cross‐sectional study (Study 4) indicated that participants who perceived the study to be more prototypical of science found it more worthwhile, reported making a wider contribution by taking part, reported less dislike for the task, more happiness at having taken part, and more trust in the researchers, all of which indirectly predicted greater followership. Implications for the theoretical understanding of obedience to toxic instructions are discussed.

## BACKGROUND

For the most part, doctors and civil servants simply did their jobs. Some merely followed orders, others worked for the glory of science. John R. Heller Jr., Director of the Public Health Service's Division of Venereal Diseases (cited in Cockburn & St. Clair, 1998, p. 67).

According to a recent conference of the European Academy of Sciences, ‘undoubtedly, one of the driving forces of the material and intellectual progress of mankind has been science and technology’ (Debru, 2018). Indeed, the right to benefit from scientific advancement is enshrined as Article 27(1) of the Universal Declaration of Human Rights.

Yet science has its dark side. Over the 3rd and 4th century BC, Herophilus is rumoured to have dissected 600 live prisoners to advance medical understanding (Von Staden & Chalcedonius, 1989). More recently, the infamous Tuskagee Syphilis Study deliberately left African American men untreated for 40 years to chart the course of the disease (Reverby, 2009). Closer to home, there is growing unease about psychological studies that explore the effects of drugs, aggressive interrogation and sustained humiliation on participants (Marks, 1991; Moreno, 2012).

Our argument is that positive and negative aspects of science are interdependent. It is the very fact that science is integral to human progress that it is vulnerable to misuse as grounds for regressive behaviour. To advance this argument we draw on recent work, which has re‐examined evidence from Milgram's (1963, 1974) ‘Obedience to Authority’ (OtA) paradigm, and which has argued that participants' willingness to harm others is contingent on their identification with the scientific cause in question (Reicher & Haslam, 2011). More specifically, we explore the claim that while participants' identification with a scientific enterprise can be positive for the scientific community and for the advancement of knowledge, it might also lead to willingness to harm others for that same cause.

As is well known, Milgram's studies examined how far people would go when instructed to harm another person (Cornwell, 2004; Milgram, 1974). Participants were told that they were taking part in important research to examine the effects of punishment on learning. They were then assigned to the role of a Teacher and asked by an Experimenter to administer a series of electric shocks escalating in 30 15‐volt intervals to a (male) Learner each time he made an error on a memory task. These shocks went all the way up to an apparently lethal 450‐volts. In reality, the Learner was an actor and the shocks were not real. Nevertheless, in the most famous ‘baseline’ variant of the studies, 26 out of 40 participants (65%) proved willing to administer the maximum level of shock (Milgram, 1974).

To explain these findings, Milgram concluded that, in the face of authority, people enter a distinct ‘agentic state’. He argued that this renders them so focused on doing the bidding of the authority that they lose awareness of the consequences of their actions. Milgram described this state as one in which ‘the individual no longer views himself as responsible for his own actions but defines himself as an instrument carrying out the wishes of others’ (Milgram, 1974, p.135). However, this account is increasingly seen as unconvincing (Blass, 2004) and has been criticized on multiple grounds (Reicher et al., 2014). In particular, there is clear evidence that, even as they continue to shock the Learner, participants are aware of, and sensitive to, the Learner's plight (Gonzalez‐Franco et al., 2018; Haslam, Reicher, & Millard, 2015; Haslam, Reicher, Millard, & McDonald, 2015; Packer, 2008).

Accordingly, researchers have recently proposed an alternative ‘engaged followership’ account of Milgram's findings (Haslam et al., 2014; Haslam & Reicher, 2012; Reicher et al., 2012). In line with this theorizing (Haslam & Reicher, 2017; Haslam et al., 2019) we use the term ‘engaged followership’ to emphasize the role of identification in followers' willingness to work towards the experimenter. In the case of obedience, this ‘engaged followership’ analysis proposes that participants' behaviour in the OtA paradigm reflects their identification with Milgram's scientific project. Here, then, ‘obedience’ increases to the extent that participants come to identify more with the science of the study and identify more with the Experimenter as a legitimate scientific authority than they with the Learner (Haslam et al., 2014; Reicher et al., 2012). Researchers have also suggested that this occurs because those who identify with a group (in this case, scientists) and its activities (science) are more likely to trust representatives of that group (scientists in positions of scientific authority) and to accept what they say as right and worthy (Haslam & Reicher, 2018).

In line with this logic, and as Milgram himself noted in his unpublished experimental notebooks (Haslam, Reicher, & Millard, 2015; Haslam, Reicher, Millard, & McDonald, 2015), the high levels of ‘obedience’ observed in his studies can be explained as deriving at least in part from the fact that participants ‘came to the laboratory to contribute to an important scientific enterprise (understanding and improving human learning) rather than to form a relationship with the Learner’ (cited in Haslam, Reicher, & Millard, 2015, Haslam, Reicher, Millard, & McDonald, 2015, p.60). As a consequence, participants trust the assurances of the Experimenter that their actions will advance that enterprise. In these terms, they obey not because they are unaware that what they are doing is wrong but because—despite their reservations—they think that what they are doing is right.

Support for this proposition has been provided by archival analysis (Haslam, Reicher, & Millard, 2015; Haslam, Reicher, Millard, & McDonald, 2015), correlational studies (Reicher et al., 2012) and experimental research (Gonzalez‐Franco et al., 2018; Haslam et al., 2014). Moreover, in a recent analysis of post‐experimental interviews, 11 out of 46 (24%) of Milgram's participants spontaneously stated that they administered the shocks because they were convinced of the experiment's importance (Hollander & Turowetz, 2017). Hence, a key factor in whether people follow an experimenter's instructions may be the extent to which they perceive that what they are being asked to do will further a cause they have committed to (i.e., in this case, scientific advancement). Certainly, this hypothesis provides an *a posteori* explanation of Milgram's own studies where ‘obedience’ dropped to 48% when he ran his experiment in commercial premises in Bridgeport (rather than science laboratories at Yale University) and to 20% when the Experimenter had no academic affiliation (Milgram, 1974).

If this argument is correct, it follows that the more participants perceive any particular study as genuinely scientific the more they will follow experimental instructions, even in tasks they consider to be aversive. Expressed differently, the more that any specific line of research is seen to be prototypical of the general category ‘science’ the more followership it should elicit.

To explore this proposition, in the current studies, we consider the role that scientific prototypicality plays in shaping followership in scientific studies. We expect that perceptions of the science as prototypical, or the extent to which a scientific endeavour is seen to represent an exemplar of the field's ideal (see Hogg & Smith, 2007), will play a key role in participants' decision to engage in tasks they might otherwise consider to be toxic. First, we predict that more prototypical sciences will result in more followership than less prototypical sciences. Second, we explore the process that leads from perceived prototypicality to followership. In line with the engaged followership model of obedience, we predict that perceived prototypicality will be associated with a number of factors likely to encourage followership, including trust in the researchers, a belief in the worthiness of the scientific cause, and feeling more at ease with taking part.

## STUDY 1

In Study 1 we test our hypothesis that research seen as more prototypical of science will result in a greater willingness to follow experimental instructions. We draw on research suggesting that sciences seen as more prototypical evoke more favourable perceptions and elicit more support for research funding than non‐prototypical sciences (Morton et al., 2006). In everyday language, this is exemplified by the distinction between the ‘hard’ science of neuroscience and the ‘soft’ science of social science. Neuroscience tends to be seen as ‘harder’, and hence, evaluated more positively and to be taken more seriously than social science (Morton et al., 2006; O'Connor & Joffe, 2013). Indeed, the public's regard for neuroscience is so strong that research, which is described with neuroscientific terminology tends to be judged more favourably than research which is not, even when its underlying logic is nonsensical (Fernandez‐Duque et al., 2015; Weisberg et al., 2008). Critically though, our analysis suggests that, as the more prototypical science, neuroscience should invoke more trust in scientists and encourage greater willingness to follow toxic experimental instructions than social science.

Using an online analogue of Milgram's paradigm involving an aversive task (assigning extremely negative descriptors to increasingly positive social groups; see Haslam et al., 2014), we, therefore, expected participants to show more followership by continuing further in a study when it was introduced as a study advancing neuroscience than when it was introduced as a study advancing social science. Furthermore, we expected an indirect effect through trust in the scientists such that participants would have greater trust in scientists when the study was introduced as a neuro‐ (rather than social) scientific study, and that this in turn would contribute to their willingness to show followership.

### Method

#### Participants and design

We recruited 198 postgraduate students from a variety of disciplines via email. Drawing from Milgram's baseline paradigm where he emphasized the scientific importance of his research (Haslam, Reicher, & Millard, 2015; Haslam, Reicher, Millard, & McDonald, 2015), the study was presented as a short project investigating prejudice and discrimination run by a postgraduate student. Hence, we relied on participants' intrinsic interest (e.g., both in the topic and in supporting a fellow postgraduate student) rather than offering a financial incentive for taking part. Participants included 120 females and 78 males ranging in age from 20 to 65 years old (*M* = 31.28, *SD* = 8.27). They were recruited from three different sites: a university in England (Site A), a university in Scotland (Site B) and from across various universities throughout the United Kingdom (Site C). The study took approximately 10 minutes to complete and a link to the online study was included in the email advertisement.

The study had a between‐participant design in which participants were randomly assigned to one of two experimental conditions: the study was either introduced as advancing a highly prototypical science (i.e., neuroscience) or a science low in prototypicality (i.e., social science). This served as our manipulation of prototypicality. To explore any potential differences across sites (see more detail below), we subjected the data to a 2 (science type: high prototypicality vs. low prototypicality) × 3 (site: A vs. B vs. C) between‐participants analysis of variance (ANOVA).

##### Ethics statement

Ethical approval was granted by the Psychology Ethics Committee at the first authors' institution at the time the study was conducted.

#### Materials and procedure

The task was based upon that used by Haslam et al. (2014). Once participants clicked on the link to begin the study, they were told that the study was being conducted either by a ‘Cognitive Neuroscience Research Group’ or by a ‘Social Science Research Group’ and they were asked to complete a manipulation check followed by three items measuring their trust in scientists.^1^ Participants read that their task was to select one word from a list of five negative adjectives (that varied across trials; e.g., deceitful, stupid, lazy, cruel, arrogant; see Katz & Braly, 1933) to describe groups depicted in a series of photographs. Following this, they were presented sequentially with 30 photographs of groups and asked to describe each one by selecting one negative adjective from this list. Images were presented, one per screen, in a predetermined sequence starting with the most unpleasant group (the Ku Klux Klan) and ending with the most pleasant (a family walking in the park).

**FIGURE 1:**
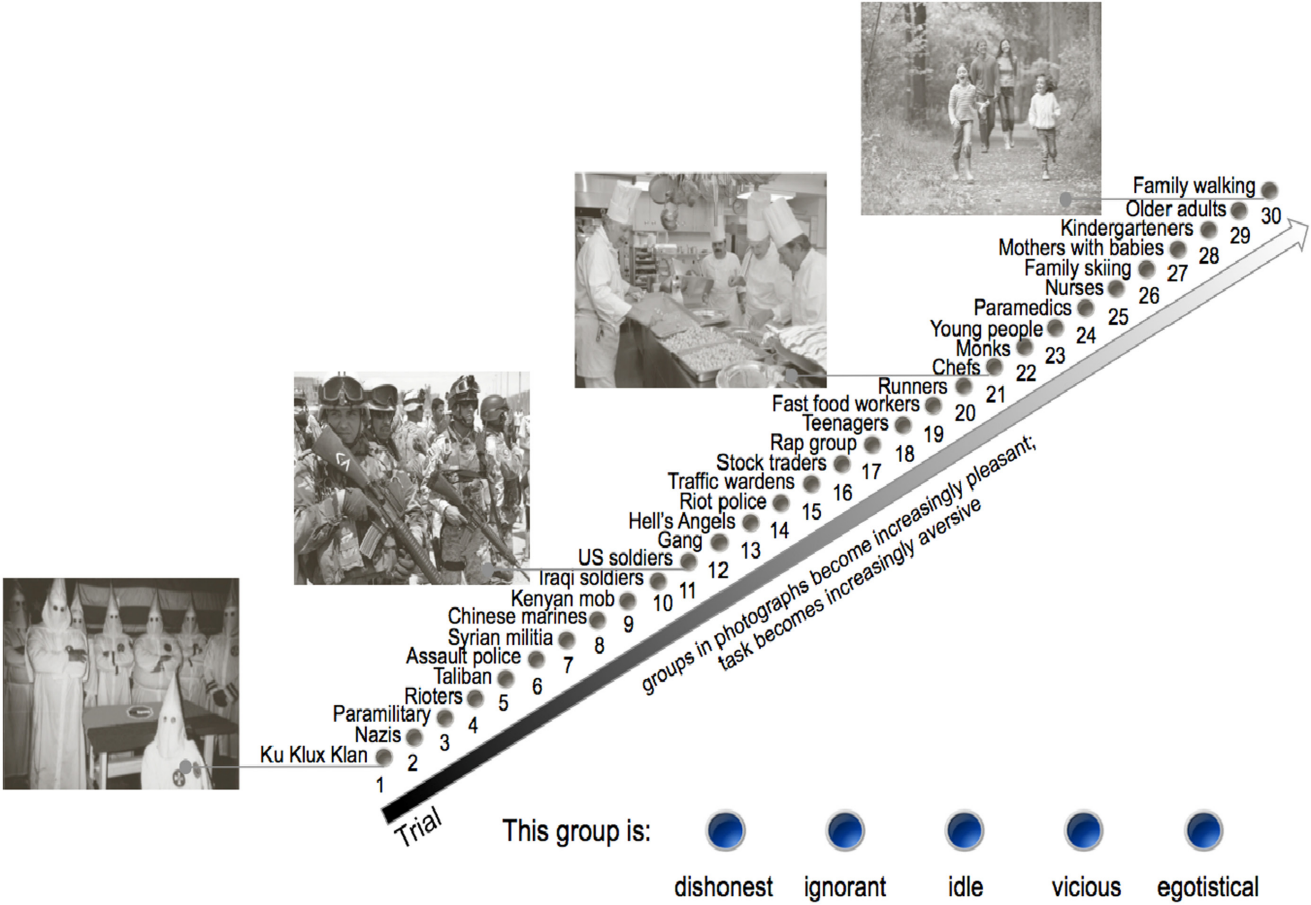
Schematic representation of the experimental paradigm.

At the bottom of each screen there were two buttons: one which read ‘Click Here to Continue’ and another, smaller button, which read ‘Stop Study’. If participants clicked the ‘Continue’ button, then a new page with the next image in the sequence came up, and the process was repeated until they pressed the ‘Stop’ button or had responded to the last (30th) picture. Because people are reluctant to describe liked groups in negative terms (Oakes et al., 1994) the task thus became increasingly aversive as the study continued (see Figure 1).

**TABLE 1:** Distribution of responses for the variable followership in studies 1–4

	Reach 30 trials	Shapiro–Wilks test	Skewness	Kurtosis
Study 1	78/198	*p* < .001	−0.490	−1.252
Study 2	269/396	*p* < .001	−2.174	4.736
Study 3	241/382	*p* < .001	−1.741	2.297
Study 4	196/306	*p* < .001	−1.714	1.943

*Note*: Reach 30 Trials = proportion of participants who completed all 30 trials.

To strengthen the manipulation, a banner was placed at the top of each page. In the neuroscience condition, the banner read ‘Cognitive Neuroscience Research Group’ and included an image of a brain. In the social science condition, the banner read ‘Social Science Research Group’ and included an image of two people talking. Once participants had either completed or withdrawn from the study they were debriefed and thanked for their time.

The study followed the same procedure across the sites. However, we made two adjustments to facilitate recruitment at each site. While the emails inviting participants to take part were identical, at one site (Site A) an additional sentence that used jargon related to neuroscience was included. At Site C, we also did not require participants to choose between moving on to the next trial or stopping the study, allowing participants to continue clicking through the paradigm (if they wished) without assigning a negative word to the picture. When analysing this data, we only included the number of trials participants actually completed. However, it became evident that many participants did not realize that this option was possible and thus, it was not part of the focal analysis. To explore whether these differences impacted on outcomes, as noted above, we included site as a variable in the analysis.

#### Measures

All measures were novel.

##### Manipulation check

To assess the perceived prototypicality of the science, we drew on the familiar distinction between ‘hard’ and ‘soft’ science. Prior to the first trial, participants in the neuroscience condition [social science condition] were asked to indicate how hard (vs. soft) they perceived neuroscience [social science] to be as a scientific discipline on a scale from 1 (soft science) to 10 (hard science).

##### Trust in scientists

Trust was measured using three items (e.g., ‘Scientists can be trusted to do the right thing’; *α* = .64). Participants responded on 7‐point scales ranging from 1 (strongly disagree) to 7 (strongly agree).

##### Followership

This was measured as the number of trials that participants completed before quitting the study (1–30).^2^

#### Pilot study

We piloted the perceived pleasantness of each picture, which determined the order that pictures were placed (see Haslam et al., 2014, for details). We also piloted how members of the public (*N* = 28) would perceive the task itself. Participants were presented with the images of Group 1 (Ku Klux Klan members), Group 10 (U.S. Soldiers), Group 20 (Chefs) and Group 30 (a family walking in the park; see Figure 1) along with the negative set of words that were used to describe each group in the main study. Using a Likert‐type scale, participants were asked to indicate (a) how toxic they would find the task in each trial (1 = not toxic at all; 7 = extremely toxic), (b) how willing they would be to engage with the task in each trial (1 = not willing at all; 7 = extremely willing) and (c) whether they believed that others would reject them for engaging in the task in each trial (1 = no, not at all; 7 = yes, definitely).

Results indicated that across these four sample trials, participants (a) perceived the task to be increasingly toxic (Ku Klux Klan: *M* = 2.43, *SD* = 1.60 < Army: *M* = 4.36, *SD* = 1.57 < Chefs: *M* = 4.39, *SD* = 1.87 < Family: *M* = 5.39, *SD* = 1.69; *F*lin = 29.17, *p* < .001) and (b) reported that they would be increasingly unwilling to perform it (Ku Klux Klan: *M* = 5.36, *SD* = 1.75 > Army: *M* = 2.07, *SD* = 1.09 > Chefs: *M* = 1.89, *SD* = 1.30 > Family: *M* = 1.61, *SD* = 0.99; *F*lin = 96.75, *p* < .001). The same pattern was evident for participants' judgement of whether others would reject them for performing the task (Ku Klux Klan: *M* = 1.86, *SD* = 1.08 < Army: *M* = 5.46, *SD* = 1.43 > Chefs: *M* = 5.04, *SD* = 1.40 < Family: *M* = 6.43, *SD* = 0.79; *F*lin = 148.02, *p* < .001).^3^

### Results and discussion

Bivariate correlations showed that followership (i.e., number of trials completed) was positively associated with both the manipulation check and with trust in scientists. The more trials participants completed, the more they rated the study's science as ‘hard’*, r*(197) = .19, *p* = .008, and the more they reported trusting scientists, *r*(197) = .22, *p* = .002.

As expected, there was a main effect of science prototypicality on perceptions of the science as ‘hard’; *F*(1, 197) = 31.42, *p* < .001, $\eta_{p}^{2}$ = .14. Participants who were told they were advancing neuroscience rated the science behind the study as ‘harder’ (*M* = 6.49, *SD* = 1.68) than those told they were advancing social science (*M* = 5.09, *SD* = 1.85, *d* = .79). This indicates that our manipulation of prototypicality had the intended effect. There was no main effect of site nor was there an interaction between site and science type, *F*s <2.28, *p*s > .110.

In terms of followership, our expectation that participants in the neuroscience condition would go further in the paradigm (*M* = 22.78, *SD* = 8.62) than those in the social science condition was also supported (*M* = 18.06, *SD* = 10.42; *F*[1, 198] = 12.73, *p* < .001, $\eta_{p}^{2}$ = .06, *d* = .49). In addition, there was a main effect of site on followership, *F*(2, 198) = 3.95, *p* = .021, $\eta_{p}^{2}$ = .04. A post‐hoc analysis of this effect indicated that participants at Site C went significantly further (*M* = 22.38, *SD* = 10.61) than those at Site A (*M* = 18.07, *SD* = 10.38; *p* = .036, *d* = .41). It is possible, then, that providing participants with the option at Site C to look through the trials may have empowered participants to complete more trials. The differences between Site A and Site B (*M* = 21.51, *SD* = 8.09), and Site B and Site C were not statistically significant, *p*s > .087.

Finally, there was a significant (albeit small) main effect of science prototypicality on trust in scientists. Participants in the neuroscience condition reported trusting scientists more (*M* = 4.70, *SD* = 0.80) than those in the social science condition (*M* = 4.46, *SD* = 1.01; *F*(1, 197) = 3.95, *p* = .048, $\eta_{p}^{2}$ = .02, *d* = .26). There was no significant main effect of site, nor was there a significant interaction between site and science type, *F*s < 1.00, *p*s > .369.

To test our hypothesis that trust in scientists would have an indirect effect on the relationship between science prototypicality and followership, we ran a bootstrapping analysis using PROCESS 3.5 Model 4 (Hayes, 2012) but this was not significant. However, there is an overall negative effect when confidence intervals are at 90%: participants in the social science condition reported less trust in scientists than those in the neuroscience condition, which was indirectly associated with them completing less trials (*b* = −.50, *SE* = .32, CI_90_[−0.456, −0.027]).

These results provide initial evidence that science prototypicality has an impact on participants' willingness to follow experimental instructions. However, the effect on trust was small and the indirect effect of trust was not statistically significant using standard 95% confidence intervals. There are two possible reasons for this. The first relates to the fact that our measure of trust was worded so as to refer to scientists in general and, therefore, may not have explained all the variation produced by our manipulation of the specific scientists who were conducting this study. The second is that trust is unlikely to be the sole factor mediating the effect of scientific authority on followership. Indeed, our theoretical analysis suggests that other factors might include participants' identification with science and with the various actors (e.g., the Experimenter, the institution) implicated in the scientific process (Haslam & Reicher, 2018; Reicher et al., 2012).

## STUDY 2

Because our participant sample in Study 1 consisted of postgraduate students (many of whom were studying science) our manipulation of prototypicality may have been particularly meaningful to them. Hence, the aim of Study 2 was to test whether the effect of science prototypicality on followership would replicate using a more general sample. Consistent with Study 1, our (pre‐registered) prediction in Study 2 was that participants in the neuroscience condition would complete more trials than those in the social science condition. In order to gauge whether neuroscience serves to enhance, or social science serves to reduce willingness to follow, we also included a control group. We expected that participants in the control group would act similarly to those in the neuroscience condition, and go further than those in the social science condition. Our logic was that in the absence of any information about the field the study was designed to advance, participants would assume that the study's science was prototypical and hence, go further in the paradigm.

### Method

#### Participants and design

A sample of 520 participants, were recruited from Prolific and paid £1.50 each for their time. Our power analysis determined that this would be sufficient for detecting an effect size as small as .40 (based on the effect found between the two experimental conditions in Study 1) with 95% statistical power. However, we did not specify that participants should have English as their native language, which, in retrospect, we realized is important considering the language level required to understand the vocabulary of the adjectives used in the paradigm. We asked participants to write about their experience with the study in an open comment box, and from this, it became clear that many did not understand the task. The first author downloaded these comments and, blind to condition, coded them for evidence of understanding. In total, the comments indicated that 396 understood the task, 36 did not, and for 88 participants, it was unclear whether they understood the task (all data are provided in the Supporting Information on the OSF project page). We decided to only include the 396 participants who we felt confident had understood the task. Of this sample, 225 were male and 170 were female (1 used an alternative description and 2 chose not to answer) and ranged in age from 18 to 75 years old (*M* = 29.60, *SD* = 11.00).

The study used a between‐subjects design with three levels. Participants were allocated to one of two experimental conditions (neuroscience vs. social science) or to a control group.

#### Materials and procedure

The procedure for participants in the experimental conditions was identical to that in Study 1. In the control group, participants took part in the same task but were not given any information about the type of science the study sought to advance. We also used the same manipulation check question and measure of followership for all three conditions.

### Results and discussion

One‐way ANOVAs were run to test the effect of science prototypicality on the manipulation check and followership. The results of the former indicated an effect of science type on perceptions that the science informing the study was ‘hard’, *F*(2, 393) = 11.23, *p* < .001, $\eta_{p}^{2}$ = .05. We had anticipated that the social science condition would be considered less hard compared to both the control and the cognitive neuroscience condition combined. Planned contrasts support our prediction; participants in the social science condition perceived the study to be significantly less ‘hard’ (*M* = 5.75, *SD* = 1.97) compared to both the cognitive neuroscience condition (*M* = 6.87, *SD* = 2.17) and the control group, *M* = 6.68, *SD* = 1.84; *t*(393) = 4.69, *p* < .001, *d* = .51.

While the means of followership by condition were in the expected direction (social science: *M* = 25.84, *SD* = 7.49; control: *M* = 26.40, *SD* = 6.24; neuroscience: *M* = 27.47, *SD* = 4.88), the main effect of condition was not significant, *F*(2, 393) = 2.26, *p* = .106. Given that we had pre‐registered our anticipation that participants who were told they were advancing neuroscience, as well as those who would were in the control group, would go further than participants told they were advancing social science, we conducted two planned comparisons: between the control group and those in the social science condition and between the two experimental conditions. Differences between the control group and the social science condition were not significant, *t*(263.34) = 1.58, *p* = .115. However, replicating findings from Study 1, results showed that participants in the neuroscience condition went significantly further with the task (i.e., showed greater followership) than those in the social science condition, *t*(202.87) = 2.03, *p* = .043, *d* = .26. Although the effect size was smaller than that found in Study 1, this pattern mirrors the earlier result and is consistent with the ‘engaged followership model of obedience’.

Although Study 2 provides evidence of the effect of perceived scientific prototypicality on followership in a more general sample, it should be noted that these results did not hold when all participants (even those who were unlikely to have understood the task) were included in the analysis. In fact, the effect was non‐existent, *F*(2, 517) = 0.016, *p* = .984. While we are confident in our exclusion criteria (see the OSF project page), ideally participants' language proficiency would be sufficient for understanding the instructions so as to avoid the need to exclude additional participants. Hence, we attempt to test the effect of science prototypicality on followership more cleanly in Study 3.

## STUDY 3

Our aim in Study 3 was to replicate the effect found in the previous two studies, where participants engaged more in followership (i.e., complete more trials) when they believed the study was advancing a more prototypical science (i.e., neuroscience as opposed to social science). We also explored how the prototypicality of the science might impact on participants' experience with the task. In line with the ‘engaged followership model of obedience’, we expected that those who believed that the science they took part in was prototypical would be more committed to the cause. Hence, we anticipated that participants in the neuroscience condition would feel more obligated to continue with the task, report less dislike for the task, perceive the study as more worthwhile, and feel happier to have taken part than participants in the social science condition.

### Method

#### Participants and design

We recruited 400 participants from Prolific, paying them £1.50 each for taking part. To avoid the issue of some participants not understanding the task that we had encountered in Study 2, we made two adjustments. First, we made it a requirement that all participants recruited for the study reported fluency in English. Second, we added several questions to assess participants' motivations for continuing by adding a short questionnaire before the debrief. As stipulated in the pre‐registration, we excluded responses from participants who ‘agreed’ or ‘strongly agreed’ with either of the following statements: ‘I did not realise I could withdraw before this point’ and ‘I got to the point that I did by mistake’. In total, 38 participants agreed with these statements leaving a final sample of 382, which was still sufficient for detecting an effect size of .40 with 98% statistical power. Our final sample ranged in age from 18 to 79 (*M* = 36, *SD* = 14.04) and included 185 males and 180 females (3 gave an alternative description and 14 chose not to answer the question).

As the means in the control condition and the cognitive neuroscience condition were similar for both the manipulation check and degree of followership in Study 2, indicating to us that participants who are not given information about the type of science the study is advancing may assume the study represents a prototypical science. Hence, we decided not to include a control group in Study 3. As a result, the study had a between‐subject design in which participants were randomly allocated to one of two experimental conditions (neuroscience or social science).

#### Materials and procedure

Participants followed the same procedure as in Study 2 with the addition of several new measures. Four items measured perceptions of the study's worthiness (e.g., ‘The study seemed worthwhile’; *α* = .82), and a further four items assessed participants' dislike for the task (e.g., ‘I found what I was being asked to do distasteful’; *α* = .87). Happiness at having taken part was measured using two items (e.g., ‘I feel good about participating in this study’; *r*[368] = .56, *p* < .001). The two items intended to measure obligation did not correlate and so were treated as distinct measures: feeling obligated to continue (i.e., ‘I felt an obligation to the researchers running the study to continue as far as I could’) and continuing for payment (i.e., ‘I was mainly interested in getting payment’). All measures used a Likert‐type scale from 1 (strongly disagree) to 7 (strongly agree).

The data was collected in December 2020 during the COVID‐19 pandemic. In this period, prior to the roll out of vaccines, changing behaviour was a prime means of limiting infection transmission. Accordingly, the science of social behaviour was receiving an increased amount of attention in the media. We anticipated that this might influence the effects of the manipulation. Therefore, we added the following measure to the manipulation check: ‘From 1 (not important at all) to 10 (very important) how important is social science (neuroscience)’? We also added an item to check our assumptions about how people perceive that neuroscience and social behaviour science: ‘From 1 (soft science) to 10 (hard science) how do you perceive neuroscience (social behaviour science) as a discipline?’ Our assessment of participants' motives for their decision to continue (or not) with the task was done by adding a brief questionnaire that was presented to them either after withdrawing or following completion. Instructions were the same for both groups (e.g., ‘we are interested in understanding why you got to the point in the study that you did’).

### Results and discussion

Means, standard deviations and correlations between all variables can be found in Table 2. In line with the previous two studies, participants in the neuroscience condition rated the science behind the study as ‘harder’ (*M* = 6.83, *SD* = 1.90) than those in the social science condition (*M* = 5.28, *SD* = 2.05; *t*[380] = 7.68, *p* < .001, *d* = .78). However, there were no differences between conditions in perceptions of the science's importance, *t*(380) = 0.67, *p* = .506. There were also no differences between the conditions on followership, *t*(380) = 0.59, *p* = .555 and participants' perceptions of the task, *t*s < 0.36, *p*s > .719.

**TABLE 2:** Means, standard deviations and bivariate correlations between variables in study 3

	*M*	*SD*	2	3	4	5	6
1. Followership (Trials)	25.73	7.05	−.35***	.08	.24***	.15**	.16**
2. Dislike for Task	3.82	1.79		−.40***	−.52***	.01	−.18**
3. Study Worthwhile	5.04	1.26			.60***	.08	−.09
4. Happy to Take Part	5.01	1.29				.07	.02
5. Obligation to Continue	5.94	1.62					−.10
6. Payment as Motivator	3.58	1.81					

*Note*: Significant correlations are indicated in bold face; *<.05, **<.01, ***<.001.

Based on the findings of our previous two studies, the lack of an effect of the manipulation on any of our DVs was surprising. Given these mixed results, it is possible that the effect is more context dependent than anticipated. As we suggested in the introduction, social science may be seen as similarly ‘scientific’ as neuroscience in the specific context of the COVID pandemic (Van Bavel et al., 2020). This possibility is supported by the finding that, while participants still reported neuroscience as ‘harder’ than social science, both were deemed equally important (neuroscience: *M* = 7.73, *SD* = 1.75; social science: *M* = 7.84, *SD* = 1.54).

If this argument is correct we would still expect, even in the context of COVID and in the absence of an effect of the neuro/social science framing, that there would be a correlation between how scientific the study is seen and the degree of followership. We address this in the fourth and final study.

## STUDY 4

In Study 4, we move away from a direct manipulation of science prototyicality.^4^ Instead, we used a cross‐sectional design to explore the processes associated with people's willingness to follow experimental instructions. Our aim was two‐fold. First, we considered the processes by which perceptions of prototypicality might lead to followership. Specifically, we anticipated that the more participants perceived that they were taking part in prototypical science, the more they would believe they were making a wider contribution to society, the more they would trust the researchers, the more they would feel the study was worthwhile, the happier they would be to have taken part, and the less dislike they would have for the task. We also expected that each of these perceptions would contribute to increased followership (i.e., completion of more trials). Second, we were interested in how the above processes would play out based on how prototypical participants believed the study as well as the study's experimenter to be. Because participants would likely make inferences about the experimenter based on the study they took part in, we expected both of these perceptions (i.e., prototypicality of the study and prototypicality of the experimenter) to be associated with the above processes.

### Method

#### Participants and design

We recruited a Prolific sample of 350 participants who were each paid £1.50 for their time. This sample was chosen based on the funds available to us. Using the same criteria for exclusion that we used in Study 3, our final sample consisted of 306 participants, sufficient to allow for stable correlation coefficients (Schönbrodt & Perugini, 2013). Of these, 181 identified as female, 123 identified as male, 1 gave an alternative description, and 1 chose not to answer this question. Participants ranged in age from 18 to 88 years old (*M* = 31.40, *SD* = 10.77).

#### Materials and procedure

Participants followed the same procedure and completed the same task as in the previous three studies with the exception that there was no manipulation or manipulation check. As in Study 3, once participants had either completed the task or withdrawn, they were asked to complete a brief questionnaire in which they reflected on their experience. We also used the same items to measure perceptions of the study's worthiness (*α* = .87), participants' dislike for the task (*α* = .89), and their happiness at having taken part, *r*(304) = .63, *p* < .001. In addition, we added perceptions of study prototypicality (e.g., ‘This study was not a serious piece of science’; recoded, *α* = .89), and perceptions of experimenter prototypicality (e.g., ‘The experimenters of this study fit my idea of what scientists are like’, *α* = .77). We also included two additional measures of process: three items measured perceptions of making a contribution (e.g., ‘By taking part in this study, I made a contribution to society’, *α* = .94), and trust in the researchers using a single item (‘I trusted the study's researchers’). All measures used a Likert‐type scale from 1 (strongly disagree) to 7 (strongly agree).

### Results and discussion

**FIGURE 2:**
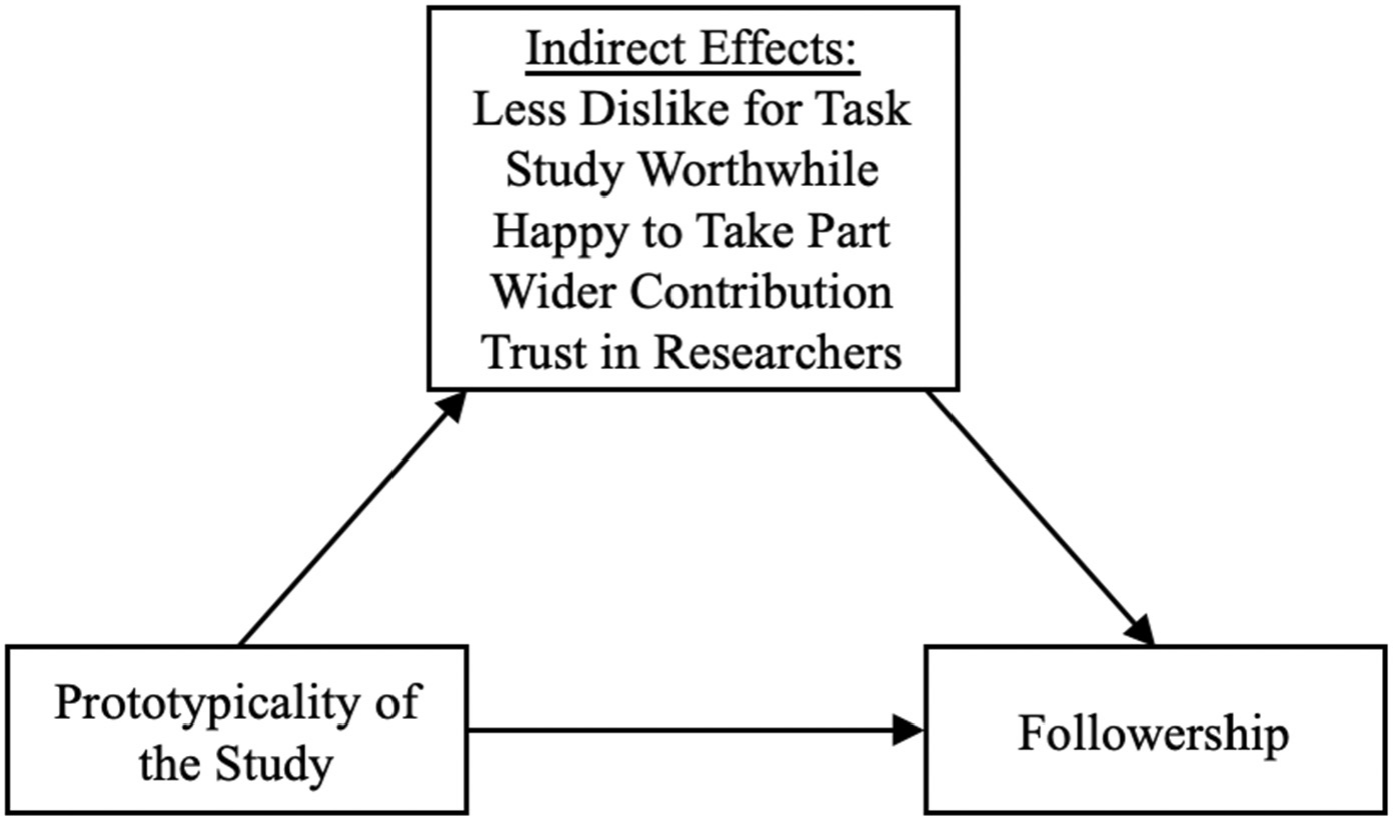
Conceptual model summarizing process analysis for the indirect effects of significant variables on the relationship between prototypicality of the study and followership.

**FIGURE 3:**
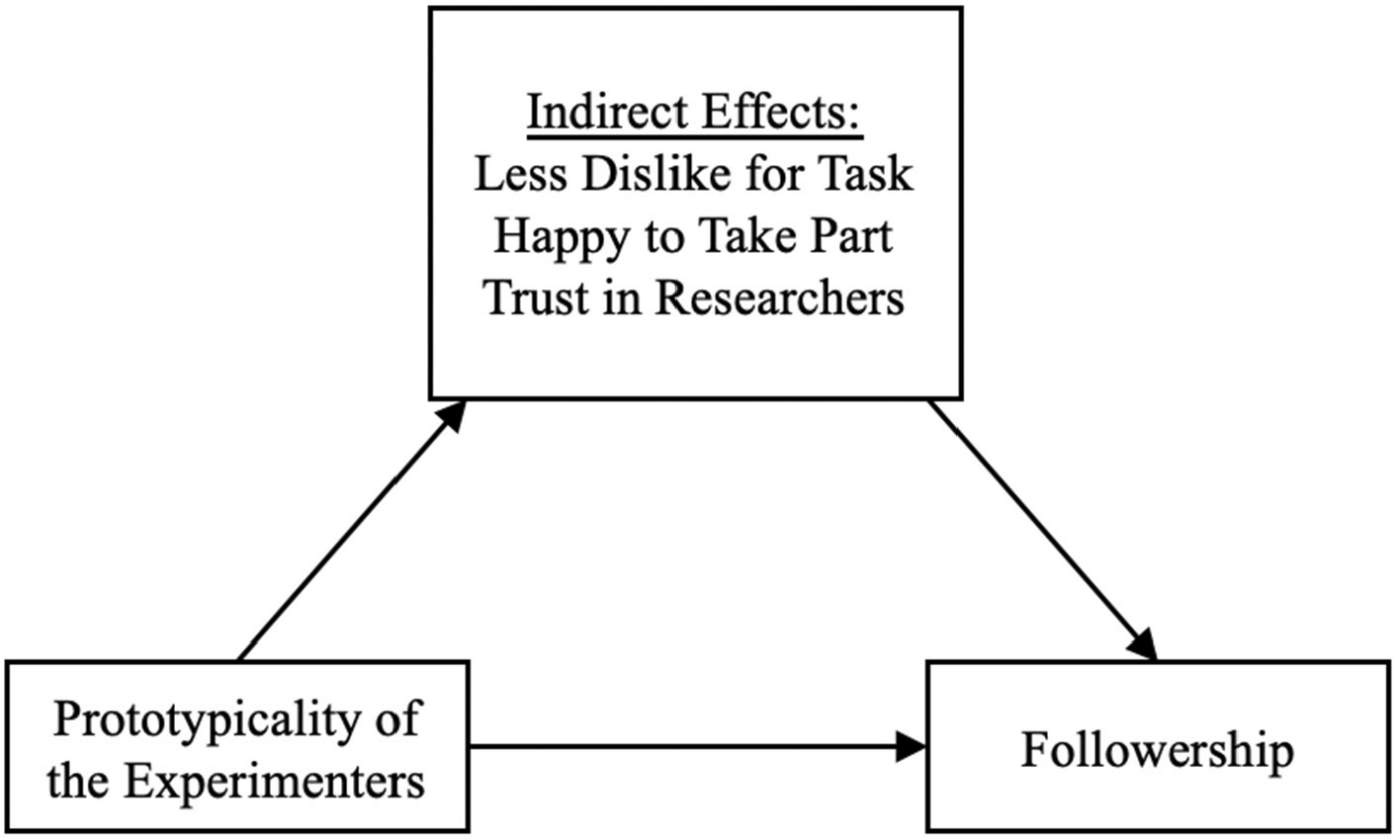
Conceptual model summarizing process analysis for the indirect effects of key variables on the relationship between prototypicality of the experimenter and followership.

Means, standard deviations and correlations between key measures are presented in Table 3. A bootstrapping analysis was conducted to examine the indirect effects of study and experimenter prototypicality on followership via the various assessed processes using PROCESS 3.5 Model 4 (Hayes, 2012). We then contrasted the indirect effect of study prototypicality with the indirect effects for experimenter prototypicality. Results for all indirect effects are reported in Table 4. A conceptual model summarizing the PROCESS analysis for each predictor variables is provided in Figures 2 and 3.

**TABLE 3:** Means, standard deviations and bivariate correlations between variables in study 4

	*M*	*SD*	2	3	4	5	6	7	8
1. Followership (Trials)	25.37	7.79	.01	.10	−.25***	.11	.25***	.11	.18**
2. Prototypicality ‐ Study	4.33	1.34		.68***	−.23***	.69***	.55***	.64***	.40***
3. Prototypicality ‐ Experimeter	4.32	1.07			−.33***	.63***	.55***	.54***	.45***
4. Dislike for Task	4.12	1.67				−.44***	−.57***	−.32***	−.37***
5. Study Worthwhile	4.67	1.34					.74***	.62***	.56***
6. Happy to Take Part	4.90	1.29						.56***	.49***
7. Wider Contribution	4.37	1.31							.48***
8. Trust in Researchers	5.18	1.40							

*Note*: Significant correlations are indicated in bold face; *<.05, **<.01, ***<.001.

**TABLE 4:** Mediators of the relationship between perceptions of prototypicality and followership—Study 4

	Indirect effect	BootSE	BootLLCL	BootULCL
Prototypicality of the study				
Dislike for Task	0.35**	0.11	0.1041	0.7171
Study Worthwhile	0.75**	0.31	0.1828	1.3814
Happy to take Part	1.13**	0.25	0.5201	1.8104
Wider Contribution	0.62*	0.27	0.1100	1.1713
Trust in Researchers	0.47**	0.17	0.0602	1.0296
Prototypicality of the experimenters				
Dislike for Task	0.58**	0.17	0.1943	1.0577
Study Worthwhile	0.33	0.35	−0.3512	1.0476
Happy to take Part	1.14**	0.31	0.3788	1.9759
Wider Contribution	0.29	0.28	−0.2371	0.9063
Trust in Researchers	0.56**	0.24	0.0076	1.2532

*Note*: Significant indirect effects are in bold face; *<.05, **<.01.

Consistent with our (pre‐registered) expectations, the results of the bootstrapping analysis (with confidence intervals at 95%), indicated that participants who perceived the study to be more prototypical reported greater trust in the study's researchers, found the study more worthwhile, reported less dislike for the task, were happier to have taken part, and felt they had made a wider contribution to society by participating. Each of these perceptions indirectly contributed to greater followership (i.e., completing more tasks).

Bootstrapping analysis (with confidence intervals at 95%) with protoypicality of the experimenter entered as the predictor indicated that participants who perceived the experimenter to be more prototypical reported greater trust in the study's researchers, were happier to have taken part, and expressed less dislike for the task, all of which were associated with them completing more trials. There were no indirect effects through perceptions of making a wider contribution or of perceiving the study as worthwhile. These results suggest that the indirect effects of study prototypicality on followership are more consistent than those of experimenter prototypicality.

## GENERAL DISCUSSION

In four studies, we examined the extent to which individuals are willing to follow experimental instructions as a function of the perceived prototypicality of a given science. Studies 1 and 2 provided evidence of a causal link whereby prototypical sciences (neuroscience, rather than social science) evoke more followership than non‐prototypical sciences. Study 3 fails to replicate the finding that a neuroscience framing produces more followership than a social science framing for reasons we explore in more detail below. In Study 4, results indicated that participants who perceived a study to be more prototypical felt that their participation made more of a contribution to society, believed more in the study's worth, reported more trust in the study's researchers, reported less dislike for what they were being asked to do, and were happier to have taken part—all of which indirectly resulted in them completing more trials. The latter three effects were also observed to the extent that participants perceived the study's experimenters as prototypical.

Together, these results provide support for a core tenet of the ‘engaged followership model of obedience’—namely that people's willingness to follow the instructions of an authority figure (in this case, an experimenter), even when they find the task they are engaging in aversive, is influenced by their beliefs about the cause they are supposedly advancing (here, the science). We found experimental evidence (Studies 1 and 2) indicating that science prototypicality enhances people's willingness to follow toxic instructions. In addition, the cross‐sectional data from Study 4 that examined issues of process revealed that perceptions of study prototypicality were associated more consistently than perceptions of experimenter prototypicality with the various examined processes. We believe this may be due to the lack of information participants had about the experimenter in this study: while they could certainly make judgements about the task they had just taken part in, they knew little about the researchers, or about the type of science they represented. Indeed, as the study took place online, participants had no direct interaction with an experimenter as they would have done in more traditional, lab‐based, studies. Although the high correlation between perceived prototypicality of the study and that of the experimenter suggests that one can be deduced from the other, it is important to investigate more thoroughly how these perceptions are derived. This would seem to be an important avenue for future research.

It is important to address the failure of our prototypicality manipulation to affect the degree of followership in Study 3. As we argued above, this may be explained in terms of the failure of our manipulation to produce different levels of perceived prototypicality rather than a failure to find a relationship between prototypicality and followership. That is, because this study was conducted eight months into the COVID‐19 pandemic people may have been more conscious of the value of social science (e.g., as argued by Jetten et al., 2020; Van Bavel et al., 2020 and evidence that people displayed generally high levels of adherence to social policies regulating people's movement; Wright et al., 2022) in ways that led them to construe it to be prototypical of good science and thereby negated our manipulation. Indeed, participants in this study deemed both neuroscience and social science as similarly important, despite the former being rated as ‘harder’ than the latter.

This possibility exposes important questions about how science is perceived by the public. In line with previous research, which found that neuroscience is perceived to be a highly prototypical science (Morton et al., 2006; O'Connor & Joffe, 2013), we had assumed that ‘harder’ sciences (that is, those evaluated most positively and taken most seriously) would be seen as more prototypical of science than softer sciences. However, it is likely more complex than this: for a science to be perceived as prototypical, it may need to be seen as having attributes such as importance, societal relevance and general usefulness, to name a few. Hence, as suggested by the results of Study 3, a science that is considered to be important may be seen as prototypical, even if it is not a ‘hard’ or a natural science. To more firmly establish the effect of prototypicality on followership, future research should investigate how the public views science in terms of the weight it places on its various attributes (e.g., as discussed by Chalmers, 2013) and how these feed into perceptions of the prototype. Along these lines, it would be worthwhile examining how people's willingness to follow instructions might be influenced by ambiguity (vs. clarity) of a prototype (Bartel & Wiesenfeld, 2013). Until we have a more nuanced understanding of how various attributes combine to underpin science prototypicality, the effects found in Studies 1, 2 and 4 should be treated with caution.

It should also be noted that the majority of participants across the four studies (61%) completed the paradigm (i.e., they did not drop out early). Given the anonymity that comes from taking part in an online study, this level of followership was not unexpected (for a discussion on the limitations of the paradigm we used, see Haslam et al., 2014). Furthermore, in Milgram's baseline study, 65% of participants completed the study (Milgram, 1974). Nevertheless, future research might consider how to increase the sensitivity of this measure as well as how it might better identify factors that may be relevant to the effect of scientific authority on followership (e.g., identification with science generally; see Haslam & Reicher, 2018). For this latter aim, qualitative analysis on why people decide to quit (vs. complete) the study might shed further light on the contribution of perceived prototypicality to followership in scientific research.

From a theoretical perspective, our results support previous evidence that people's willingness to follow an authority figure's instructions is not an act of blind obedience as postulated by Milgram's (1974) ‘agentic state’ account. Instead, their ‘obedience’ is at least partly dependent on their perceptions of the cause they believe they are advancing. Taken together, the present research builds on previous work in suggesting that ‘obedience’ is shaped, at least in part, by followers' perceptions of the means being used to further a cause they have committed to supporting (in this case, the science they believe the study is contributing to). While research is needed to solidify the specific effects of perceived prototypicality on followership, the evidence here supports an ‘engaged followership model of obedience’, which argues that people may be willing to put concerns they might have about a task aside if they perceive that task to be aligned with, and furthers, a worthy cause.

From a practical perspective, we hope that this research draws attention to the susceptibility of science to the potential for misuse in its name. While more work is needed to understand how and why people continue with scientific tasks they consider to be aversive, the evidence presented here should serve as a reminder to scientists of their potential to influence those who take part in their endeavours. While the scientific community has made great strides in recent years to promote ethical practices and transparency, we must remain vigilant about how science might be misused in its name.

## AUTHOR CONTRIBUTIONS

**Megan E. Birney:** Conceptualization; data curation; investigation; project administration; writing – original draft; writing – review and editing. **Stephen D Reicher:** Conceptualization; funding acquisition; methodology; project administration; supervision; writing – original draft; writing – review and editing. **S Alexander Haslam:** Conceptualization; methodology; project administration; writing – original draft; writing – review and editing. **Niklas K Steffens:** Formal analysis; writing – review and editing. **Fergus G. Neville:** Formal analysis; funding acquisition; methodology; writing – review and editing.

## CONFLICT OF INTEREST

The authors have no conflicts of interests to declare.

## Data Availability

The materials used and the data reported are publicly accessible and can be found here: https://osf.io/nk3a9/?view_only=d45189cc4d2147c989672baf32582f28

## Footnotes

^1^
Prior to beginning the paradigm, participants indicated their general ideas about science, whether they regarded the study as important, and whether they valued volunteering for scientific research generally. These were exploratory and the items did not measure what they were intended to. There was also an exit questionnaire designed to assess particpant's experience of the study. However, because many participants dropped out of the study, there was not sufficient data to warrant analysis. It is also important to note that these studies were not pre‐registered, unlike the three subsequent studies reported in this paper.

^2^
Due to the nature of this variable (with a ceiling of a maximum score of 30), the data is not normally distributed (see Table 1 for the distribution of responses across all four studies). Based on our sample size and the research indicating that both parametric and non‐parametric tests may be appropriate (Poncet et al., 2016; Wadgave & Khairnar, 2019), we chose to analyse our data using parametric tests.

^3^
Compared to chefs (who have been shown to be the more pleasant group), participants reported that using negative adjectives to describe U.S. soldiers would be slightly more likely to result in rejection from others. We attribute this to the fact that it is controversial to be critical of the military generally, particularly from one's own country or that of a close ally.

^4^
Prior to this study, we sought to develop a new manipulation of prototypicality by creating a ‘messy’ version of the paradigm as a non‐prototypical condition, which included numerous typos and commands throughout. This is pre‐registered as Study 4 of this project. However, after pilot testing (*N* = 60) it became clear that the inclusion of errors was not associated with perceptions of prototypicality. We, therefore, decided to drop the creation of this new manipulation in Study 4 and conducted a cross‐sectional study. Hence the Study 4 reported in this paper corresponds to pre‐registered ‘Study 5’ on the project's OSF page.
